# Notice of Retraction and Replacement. Chaarani B, et al. Association of Video Gaming With Cognitive Performance Among Children. *JAMA Network Open*. 2022;5(10):e2235721.

**DOI:** 10.1001/jamanetworkopen.2023.6895

**Published:** 2023-04-10

**Authors:** Bader Chaarani, Joseph Ortigara, DeKang Yuan, Hannah Loso, Alexandra Potter, Hugh P. Garavan

**Affiliations:** 1 Department of Psychiatry, University of Vermont, Burlington

**To the Editor** We write to respond to concerns raised about the analyses and findings for the Original Investigation, “Association of Video Gaming With Cognitive Performance Among Children,” published in *JAMA Network Open* on October 24, 2022.^1^ These concerns were brought to our attention by a reader, for which we are grateful.

This study was conducted to examine the association between video gaming and cognition in children using data from the Adolescent Brain Cognitive Development (ABCD) study. Cognitive performance and blood oxygen level–dependent (BOLD) signal were compared in video gamers (VGs) and non–video gamers (NVGs) during response inhibition and working memory using task-based functional magnetic resonance imaging (fMRI) among children from the ABCD study ages 9 and 10 years. We collected data on demographic characteristics and mental health measures using the Child Behavior Checklist (CBCL) and attempted to control for confounding of these factors. However, the 2 groups were different in terms of sex, race and ethnicity, parental income, and some mental health and behavioral scores. We had erroneously reported this study as a case-control study, but it should have been reported as a cross-sectional study.

The first set of errors are in Table 1.^1^ Due to inconsistent use of lists of participants and nuisance covariates by different authors, Table 1 included inaccurate information on the reported variables in terms of means, SEs, percentages and *P* values (when applicable).^1^ With the correct participants and covariates, all of the data in Table 1 have now been corrected. The data in Table 2 were correct in the original article.^1^

To compare the 2 groups on demographic factors (eg, age, sex, race and ethnicity, household income) and scanner manufacturer, we used *t* tests and χ^2^ analyses consistent with our original study. To compare the 2 groups on IQ, body mass index, mental health, and other cognitive tasks, our reanalysis used appropriate linear mixed models, which controlled for sociodemographic factors (age, sex, puberty, race and ethnicity, and household income). To compare task fMRI performance metrics between the 2 groups, we use the same model with IQ and scanner site added as an additional covariate. These covariates were used in the original study for the neuroimaging analyses and are now consistently applied to other group comparisons. All of the analyses in question have been repeated, with the involvement of 2 scientists who did not perform the initial analyses, and whom we thank in the Additional Contributions. Our main conclusions do not change, and we still find that “compared with NVGs, VGs were found to exhibit better cognitive performance involving response inhibition and working memory as well as altered BOLD signal in key regions of the cortex responsible for visual, attention, and memory processing.”^1^ However, there were other important errors, and some of the key findings do change and are more completely reported here and in the corrected version of the article.

The second error involves our original reporting of race and ethnicity, which we erroneously reported as not being included in the ABCD study data. In our corrected analyses, race and ethnicity information are now included in all of the linear mixed models comparing the 2 groups. To adhere to the journal’s publication guidelines, we have also added the race and ethnicity breakdown for each sample in the corrected Table 1.^1^

The third error involves differences between the 2 groups. Originally, we had stated that “mental health and behavioral scores from the CBCL were not significantly different between NVGs and VGs.” After correction for multiple testing using false discovery rate (at *P* < .05), attention problems, depression symptoms, and attention-deficit/hyperactivity disorder scores were significantly higher among VGs compared with NVGs. This has been corrected in the Abstract, Results, and Conclusions sections and in Figure 2.^1^

The fourth error occurred in Supplement 1, in which we had erroneously stated that the VG group outperformed the NVG group on the Youth National Institutes of Health sorting task and the Rey Auditory Verbal Learning Test (RAVLT).^1^ The findings from our reanalyses show that the NVG group significantly outperformed the VG group on the list sorting task and there was no significant difference on the RAVLT between the 2 groups.

The fifth set of errors pertain to the reported reaction times. We have corrected the reporting of variance for the mean reaction times from SD to SE. In addition, we had not indicated that the millisecond differences in reaction times between the 2 groups, although statistically significant, were small, and this has now been noted in Abstract and main text of the corrected article.

We apologize to the readers and editors of *JAMA Network Open* for any confusion this may have caused. Given the pervasiveness of the errors in our analyses and original findings, the editors have requested that the original article be retracted and replaced with a corrected article. The corrections affect the Key Points, Abstract, main text, Table 1, Figure 2, and Supplement 1.^1^ The replacement article includes new supplements with a copy of the original article with the errors highlighted and another copy with the corrections highlighted.^1^
